# Easily detected signs of perineural tumour spread in head and neck cancer

**DOI:** 10.1007/s13244-018-0672-8

**Published:** 2018-11-16

**Authors:** Jan Willem Dankbaar, Frank A. Pameijer, Jeroen Hendrikse, Ilona M. Schmalfuss

**Affiliations:** 1 Department of Radiology, University Medical Center Utrecht, (HP E01.132), PO Box 85500, 3508 GA Utrecht, The Netherlands; 2 Department of Radiology, University of Florida College of Medicine, Gainesville, FL USA

**Keywords:** Head and neck, Oncology, Perineural tumour spread, Imaging

## Abstract

**Abstract:**

Perineural tumour spread (PNTS) in head and neck oncology is most often caused by squamous cell carcinoma. The most frequently affected nerves are the trigeminal and facial nerves. Up to 40% of patients with PNTS may be asymptomatic. Therefore, the index of suspicion should be high when evaluating imaging studies of patients with head and neck cancer. This review describes a “quick search checklist” of easily detected imaging signs of PNTS.

**Teaching Points:**

• *A distinctive feature of head and neck tumours is growth along nerves.*

• *Perineural tumour spread is most often caused by squamous cell carcinoma.*

• *There are several key findings for the detection of perineural tumour spread.*

## Introduction

Head and neck cancer accounts for 5–10% of all cancers in the United States and Europe [1]. Like other malignancies, head and neck cancer may spread through direct extension and/or via haematogenous or lymphatic spread. However, a distinctive feature of head and neck tumours is growth along nerves [2]. This growth is referred to as perineural tumour spread (PNTS) and perineural invasion. These two types of nerve involvement are distinct on pathological examination, but cannot be differentiated from each other by imaging. Therefore, PNTS will be used throughout the text for both types of nerve involvement. In PNTS patients, tumour cells follow the nerve using the epineurium, perineural and endoneural spaces as a path of least resistance to disseminate away from the primary tumour site [3, 4]. This can occur in an antegrade and retrograde fashion.

Certain tumour types place the patient at significantly increased risk for development of PNTS. Adenoid cystic carcinoma is well known for its propensity for PNTS [5]. Adenoid cystic carcinoma most commonly affects the major salivary glands but may also arise in minor salivary glands. Minor salivary glands are dispersed throughout the entire head and neck area but are especially high in concentration in the palate [6], buccal mucosa, parapharyngeal space, paranasal sinuses and nasal cavity [7]. In addition, rhabdomyosarcomas [8], melanomas [9] and extra-nodal lymphomas have a high incidence of PNTS; however, all these tumours are rare and therefore account only for a minority of PNTS cases. Although squamous cell carcinoma has a low prevalence for PNTS, it is responsible for the majority of PNTS cases as this tumour type represents the vast majority of head and neck cancers. Especially patients with cutaneous squamous cell carcinoma are at risk for development of PNTS.

The trigeminal and facial nerves are the nerves that are most commonly involved in PNTS. PNTS along these nerves may result in facial pain, facial numbness and/or weakness of muscles supplied by these nerves. These symptoms are, however, not specific for PNTS and can be related to “benign” disease processes such as schwannoma, ischaemic or haemorrhagic stroke, neuritis, sarcoidosis, vascular compression syndrome, facial nerve haemangioma or temporomandibular joint disorders [8, 10]. In addition, up to 40% of patients with PNTS may be asymptomatic despite gross spread of disease on imaging and/or histopathology. Therefore, it is critical that the radiologist searches for potential pathology along the trigeminal and facial nerves in every patient, even in those who undergo imaging for unrelated reasons such as non-specific headaches, sinusitis or neck pain. In such instances, the imaging study will not be optimised to a specific cranial nerve and the trigeminal or facial nerve pathology might be overlooked by the interpreting radiologist. In case of PNTS, this may delay the diagnoses and subsequent treatment, consequently leading to an unfavourable outcome. Complete mapping of PNTS spread can be challenging, especially on non-focused imaging studies. In 1998, Curtin pointed out that obliteration of certain fat pads can indicate the presence of PNTS [11]. Another important clue to PNTS is denervation of certain muscles. Often, both clues can be easily appreciated on routine, non-focused, cross-sectional imaging studies and alert the radiologist to possible trigeminal or facial nerve pathology requiring a more focused imaging study that better clarifies the cause and extent of the fat pad obliteration and/or muscular denervation.

This paper describes the anatomy of the trigeminal and facial nerves as well as pertinent branches, focusing on fat pads along their course and muscles innervated by these nerves. Computed tomography (CT) and magnetic resonance imaging (MRI) examples of normal and abnormal appearance of each fat pad and of muscular denervation (acute to chronic stages) are given in different planes. A “quick search checklist” is provided to facilitate detection of facial and trigeminal nerve pathology even by non-subspecialised radiologists (Table 1). This checklist emphasises the imaging plane that best demonstrates the pertinent anatomical landmark.

**Table 1:** Quick search checklist

Fat pad or muscle signs	Cranial nerve affected	Imaging plane(s) best seen on
Fat pad anterior to the superior orbital rim	V1	Axial and sagittal
Fat superior to the levator palpebrae muscle	V1	Coronal and sagittal
Preantral and postantral fat pads	V2	Axial
Fat in pterygopalatine fossa	V2	Axial and coronal
Fat pad anterior to the mental foramen	V3	Axial
Mandibular bone marrow fat (in older adults ONLY, after red bone marrow has converted to fatty bone marrow)	V3	Axial and coronal
Fat pad at mandibular foramen	V3	Axial and coronal
Muscle of mastication	V3	Axial and coronal
Fat pad at stylomastoid foramen	VII	Axial

## Anatomy and pathology

### Trigeminal nerve

The trigeminal nerve is the largest of the cranial nerves. Its sensory functions include the general sensibility of the face, paranasal sinuses and oral cavity. Its motoric branch provides innervation to the muscles of mastication. From its brain stem nuclei, the main trunk of the trigeminal nerve emerges from the lateral pons to enter the trigeminal cistern (i.e. Meckel’s cave). Meckel’s cave contains the Gasserian ganglion. Distal to the Gasserian ganglion, the trigeminal nerve trifurcates into the three principal branches: the ophthalmic (V1), the maxillary (V2) and the mandibular (V3) nerves.

### Ophthalmic nerve (V1)

**Fig. 1:**
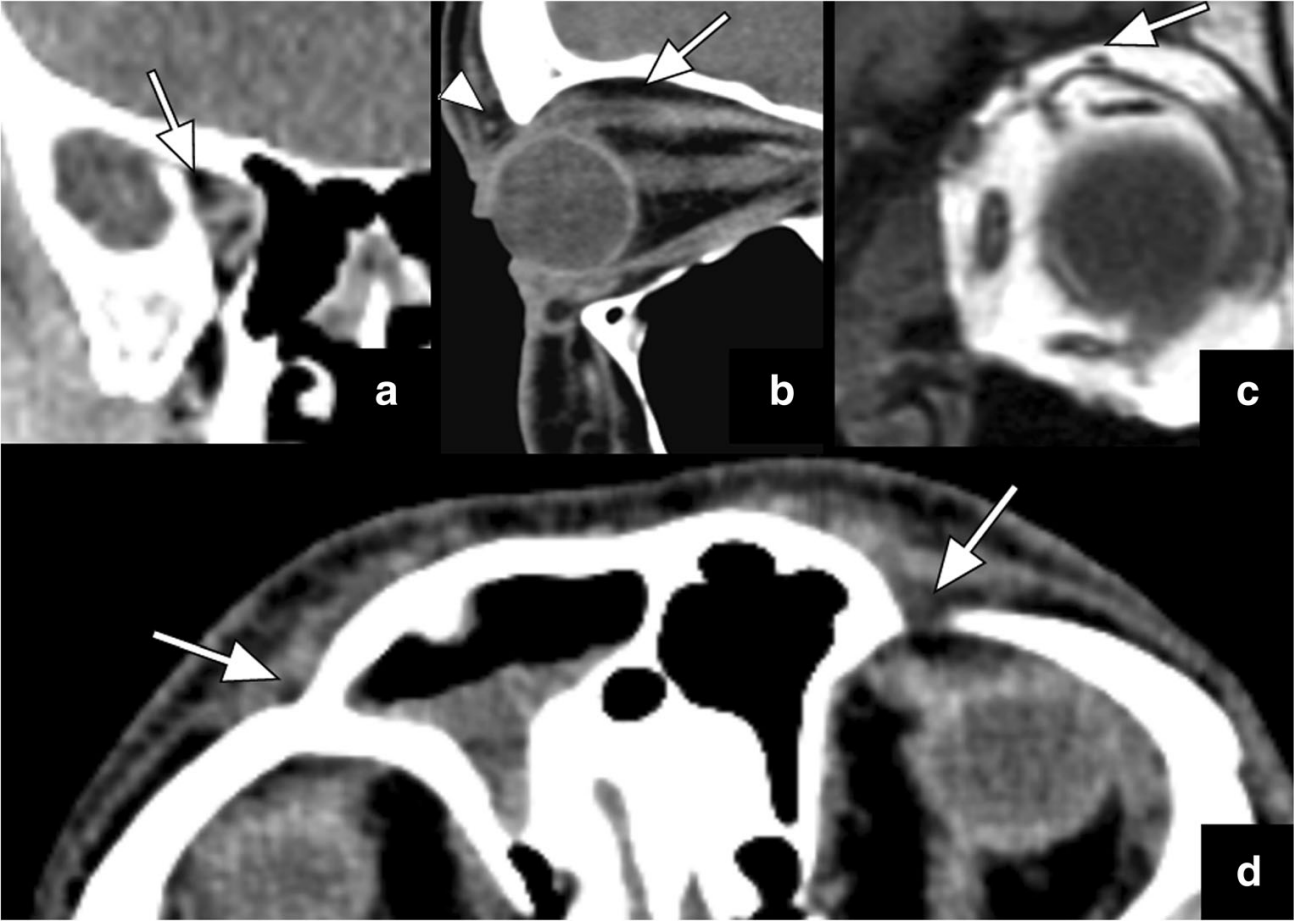
Coronal (**a**) and oblique sagittal (**b**) reformatted CT images, as well as coronal (**c**) T1-weighted images illustrate the normal fat pad (*arrows*) around the ophthalmic nerve (V1) at the orbital apex (**a**) and superior to the levator palpebrae muscle (**b**–**c**). Axial CT image (**d**) demonstrates small normal fat pads anterior to the superior orbital rim (*arrows*) which can be also well-appreciated on (**b**) the oblique sagittal CT (*arrowhead*)

V1 is the first division of the trigeminal nerve. It travels in the lateral wall of the cavernous sinus to exit the skull base through the superior orbital fissure at the orbital apex. A small fat pad is typically seen within the orbital apex that can be routinely visualised on head, neck and sinus CT and MRI studies (Fig. 1a). Within the orbit, V1 continues anteriorly within the fat pad along the orbital roof located superior to the superior rectus and superior levator palpebrae muscles (Fig. 1b–c). It then subdivides into three separate nerves: the lacrimal nerve, the frontal nerve and the nasociliary nerve. The frontal nerve continues anteriorly through the supraorbital notch to subdivide into small branches within a small fat pad anterior to the superior orbital rim (Fig. 1b and d).

**Fig. 2:**
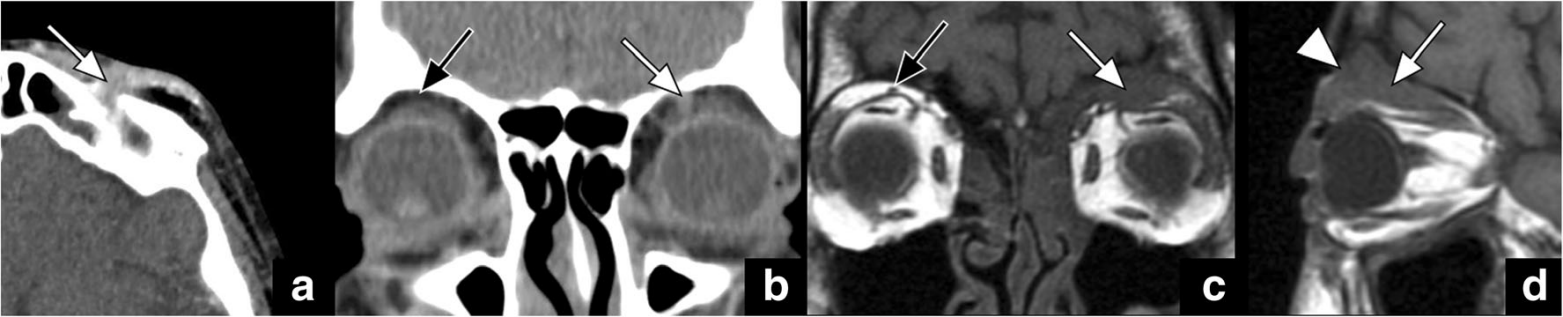
Axial CT (**a**) and coronal reformatted CT (**b**) images show obliteration of the fat pad anterior to the superior orbital rim (*arrow* in **a**) and superior to the levator palpabrae muscle (*white arrow* in **b**) when compared to its normal appearance (*black arrow* in **b**). The coronal (**c**) and sagittal (**d**) T1 weighted images in a different patient reveal obliteration of the fat pad superior to the left levator palpebrae muscle (*white arrows* in **c** and **d**) in the expected location of V1, compared to the normal fat pad on the right side (*black arrow* in **c**). Notice also the obliteration of the fat pad anterior to the supraorbital foramen (*arrowhead* in **d**). These signs are indicative of PNTS along V1 in both of these patients with a history of excised skin cancer on the forehead and V1 numbness

On imaging studies, fat should be clearly visible in the orbital apex (best seen in coronal and axial plane), along the roof of the orbit (best seen in sagittal and coronal plane), and anterior to the superior orbital rim (best seen in axial and sagittal planes) (Fig. 1). Obliteration of these fat pads, even on non-dedicated CT and MRI studies, may be the first hint for underlying V1 pathology such as PNTS (Fig. 2) and should lead to further work-up with optimised imaging protocols.

### Maxillary nerve (V2)

**Fig. 3:**
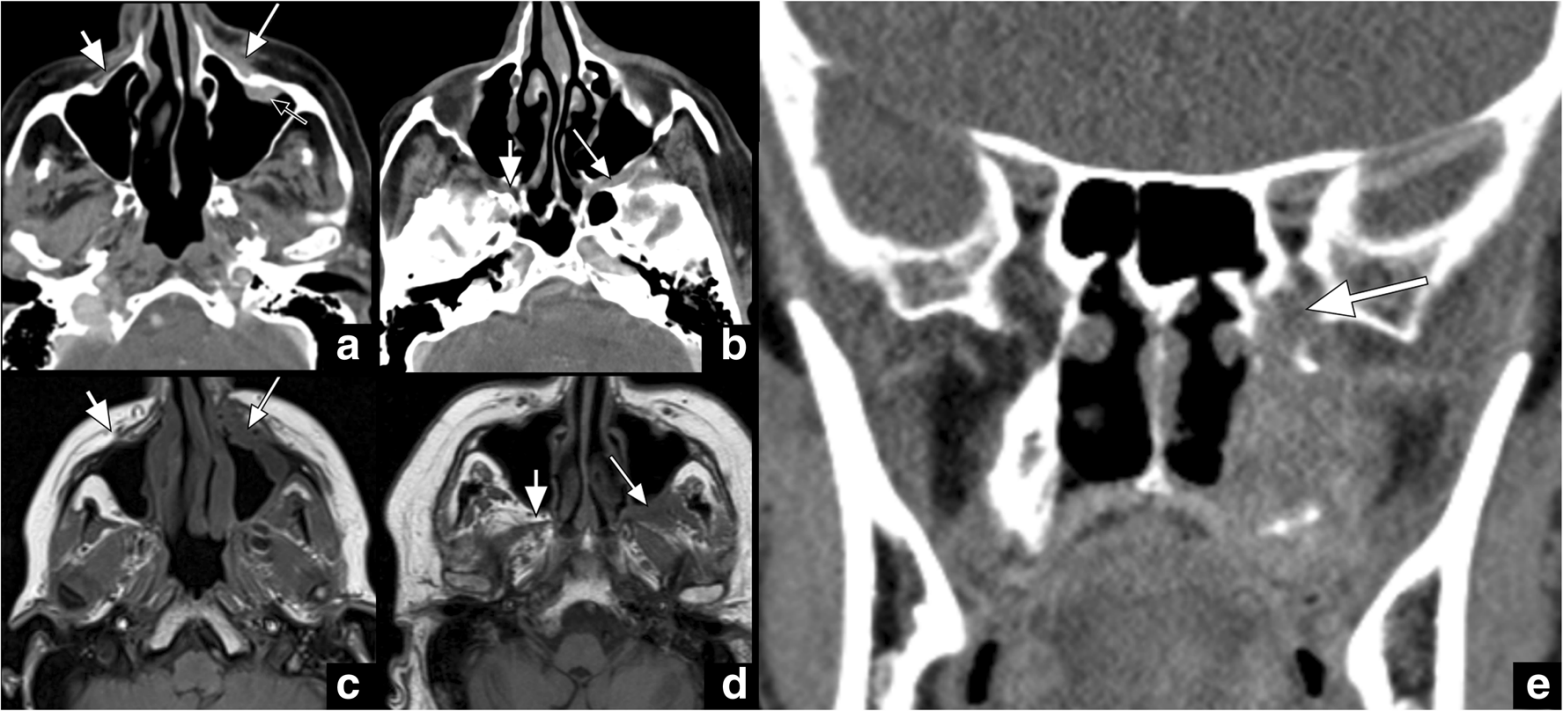
Axial CT (**a** and **b**) and axial T1-weighted (**c** and **d**) images show obliteration of the preantral fat pad on the left (*long white arrow* in **a** and **c**) when compared to its normal appearance on the right (*short white arrow* in **a** and **c**). In addition, note the enhancing soft tissue within the maxillary sinus (*black arrow* in **a**) as well as the obliteration of the fat pad in the left pterygopalatine fossa (*long white arrow* in **b** and **d**) compared to its normal appearance on the right (*short white arrow* in **b** and **d**). The obliteration of the fat pad in the left pterygopalatine fossa (*long white arrow*) can also be well appreciated on coronal images (**e**). These signs are indicative of PTNS along V2 in this patient with history of left cheek skin cancer presenting with V2 numbness

V2 also courses along the lateral margin of the cavernous sinus just beneath V1 to exit the skull base through the foramen rotundum. After passing through the foramen rotundum, it enters the cephalad portion of the pterygopalatine fossa (PPF), giving off several branches, including the zygomatic, pterygopalatine, superior alveolar and palatine nerves. The PPF contains a prominent fat pad that is best visualised in the axial (Fig. 3) and coronal planes. The main trunk of V2 continues first laterally within the retroantral fat pad and subsequently anteriorly as the infraorbital nerve along the floor of the orbit within the infraorbital canal. The infraorbital nerve emerges into the face through the infraorbital foramen that is surrounded by a small fat pad that is also referred to as the preantral fat pad (Fig. 3). The preantral and retroantral fat pads are best visualised in the axial plane. Obliteration of any one of these fat pads needs to raise suspicion for V2 pathology such as PNTS (Fig. 3) and requires further evaluation with a dedicated study.

### Mandibular nerve (V3)

**Fig. 4:**
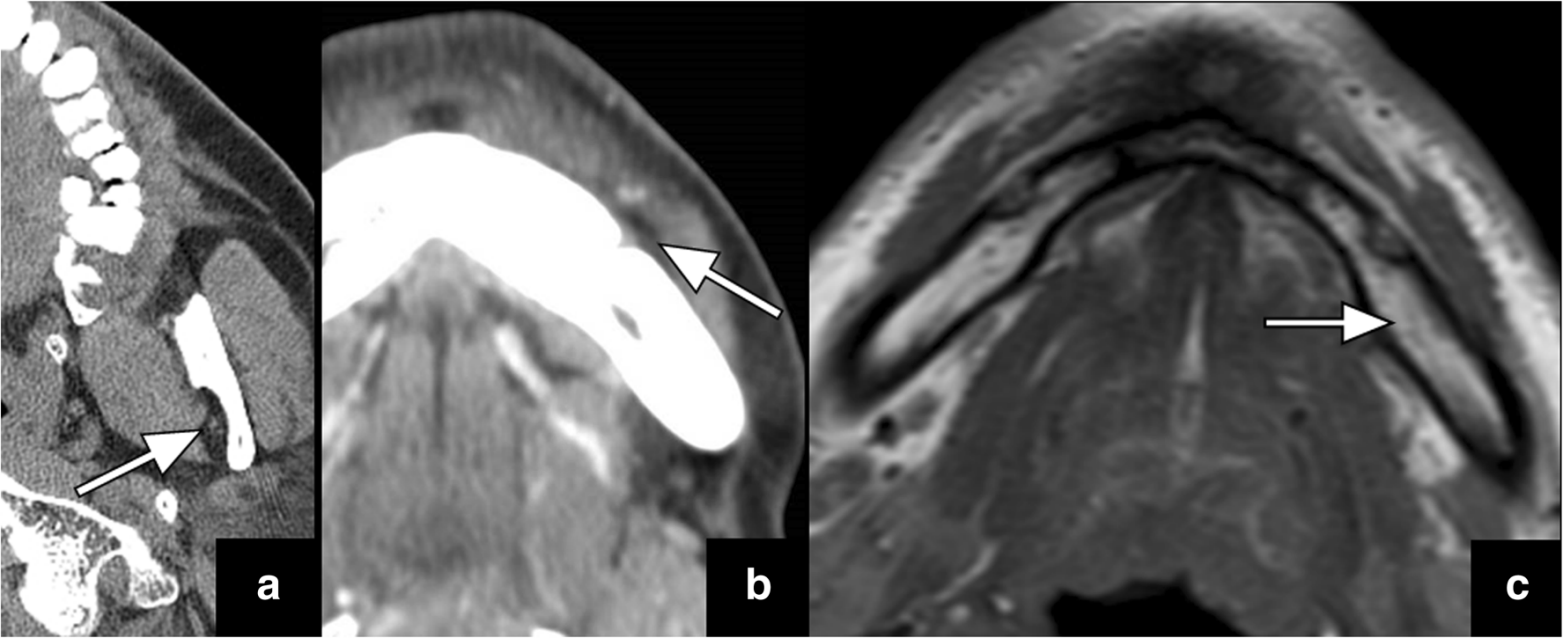
Axial (**a** and **b**) CT images demonstrate a normal prominent fat pad at the mandibular foramen (*arrow* in **a**) as well as a markedly smaller fat pad anterior to the mental foramen (*arrow* in **b**). Axial T1 weighted image illustrates the normal fatty bone marrow in the mandible (*arrow* in **c**) that is typically observed in older adults, after red bone marrow normally has converted to fatty bone marrow

**Fig. 5:**
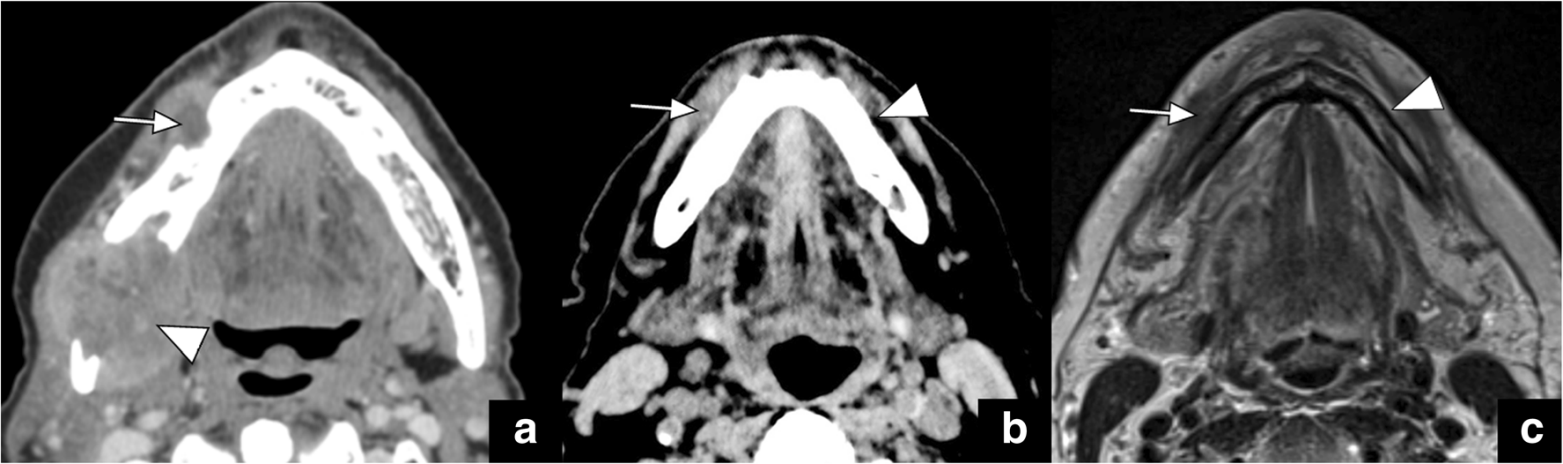
Axial CT image (**a**) in a patient with lower face numbness reveals obliteration of the fat pad at the right mental foramen at the expected location of the mental nerve (*long arrow* in **a**) with a large primary tumour seen at the right retromolar trigone (*arrowhead* in **a**). This is an example of antegrade PNTS. Axial CT (**b**) and T1-weighted (**c**) images in a different patient illustrate a more subtle obliteration of the fat pad anterior to the right mental foramen (*arrow* in **b** and **c**). This becomes more apparent when a comparison to the normal left-sided fat pad (*arrowheads* in **b** and **c**) is made. This patient was asymptomatic and had a history of lip cancer removal

**Fig. 6:**
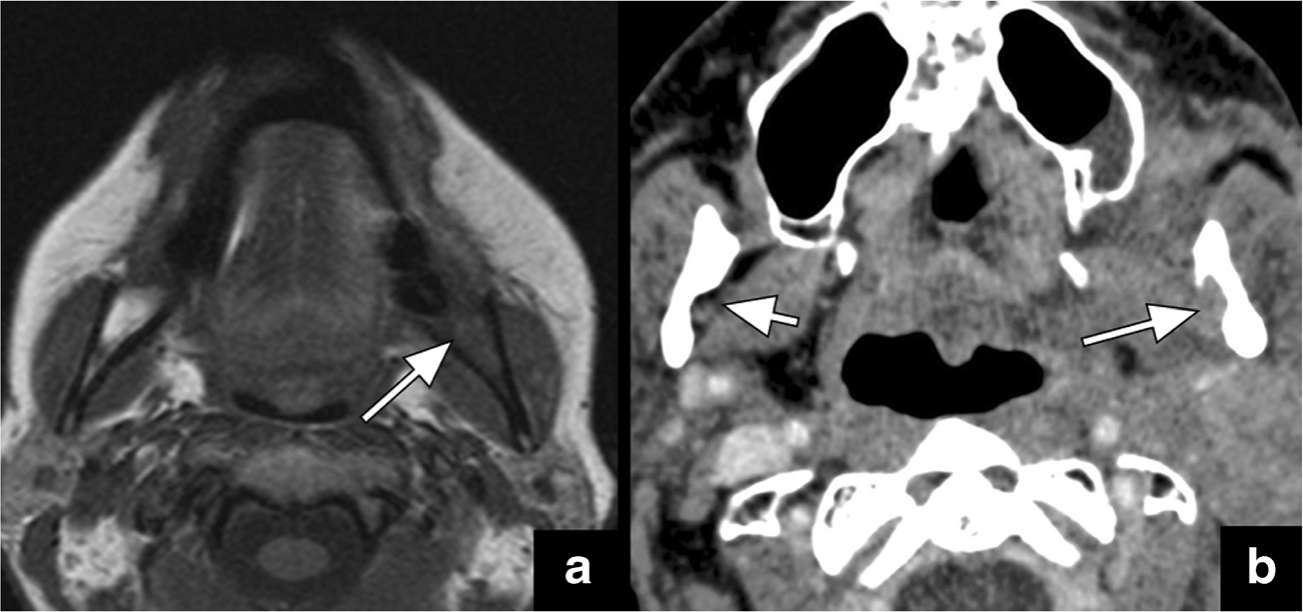
Axial T1-weighted image (**a**) shows obliteration of the fatty bone marrow in the left mandible (*arrow* in **a**). The axial CT image (**b**) of the same patient reveals obliteration of the fat pad at the mandibular foramen (*long arrow* in **b**), compared to the normal fat pad on the right (*short arrow* in **b**). These signs are indicative of PTNS along V3 in this patient with history of gingival cancer and V3 numbness

V3 is the only trigeminal nerve branch bypassing the cavernous sinus and carrying both sensory and motor fibres. The sensory fibres relay within the trigeminal ganglion along the floor of the trigeminal cistern, while the motoric branch runs beneath the ganglion to coalesce with the sensory fibres before they exit the skull base through the foramen ovale. After passing through the foramen ovale, V3 enters the masticator space and divides into the following sensory branches: the buccal, auriculotemporal, inferior alveolar and lingual nerves [12]. The inferior alveolar nerve enters the mandibular foramen on the medial side of the ramus and runs through the inferior alveolar canal to exit at the chin level through the mental foramen as the mental nerve. A prominent fat pad is usually visualised at the mandibular foramen that is best seen in the axial and coronal planes (Fig. 4a). Only a small fat pad is usually observed anterior to the mental foramen that is best displayed in the axial plane (Fig. 4b). In addition, the inferior alveolar canal is surrounded by fatty bone marrow in older patients (Fig. 5c), while red bone marrow is usually observed in children and young adults. Obliteration of any of these fat pads may be a sign of V3 pathology such as PNTS (Figs. 5 and 6) and requires further evaluation with a dedicated study to narrow the differential diagnosis and determine the extent of the disease. Malignant masses can also exhibit direct bone invasion with accompanying bone marrow changes.

**Fig. 7:**
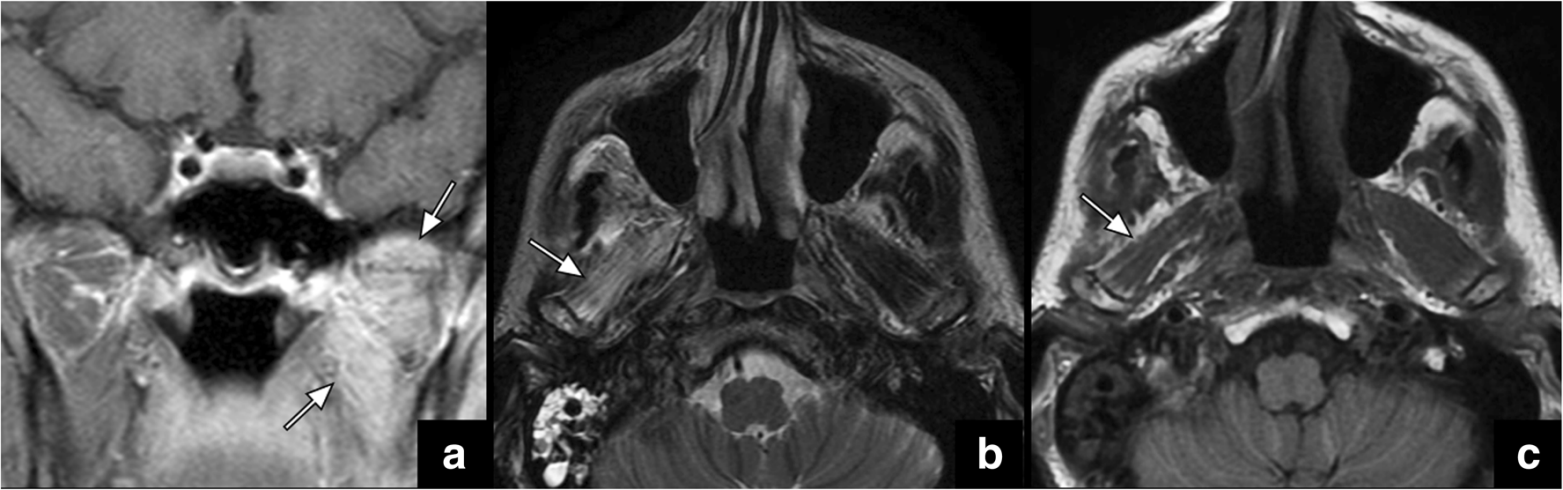
Coronal contrast-enhanced T1-weighted image (**a**) shows marked enhancement of left pterygoid muscles (*between arrows* in **a**) indicative of acute denervation of the muscles supplied by the motoric branch of V3. On an axial T2 weighted image in a different patient (**b**), the denervated muscles show marked hyperintensity (*arrow* in **b**) due to oedema in the acute phase and due to fatty replacement in the chronic phase. Muscular volume loss is usually the only distinguishing feature between the two on T2-weighted images with atrophy present in the chronic phase. Muscular atrophy and fatty replacement, as reflection of chronic denervation, can also be easily identified on axial T1 sequence (*arrow* in **c**). These findings can indicate the presence of (motoric) V3 malfunction due to PNTS

The motoric masticator nerve supplies the muscles of mastication: the masseter, temporalis, as well as the medial and lateral pterygoid muscles. The mylohyoid nerve, which is a branch of the inferior alveolar nerve, provides motor innervation to the mylohyoid and anterior belly of the digastric muscle along the floor of mouth. Disease processes affecting V3 may therefore manifest with denervation of the above muscles. In the acute phase (< 1 month), the affected muscles show oedema and enhancement that is usually best visualised on MRI (Fig. 7a, b). In the chronic phase (> 6 months), fatty infiltration and atrophy of the involved muscles can be observed on CT and MRI (Fig. 7c) [13]. This can be best appreciated in the axial and coronal planes.

### Facial nerve

**Fig. 8:**
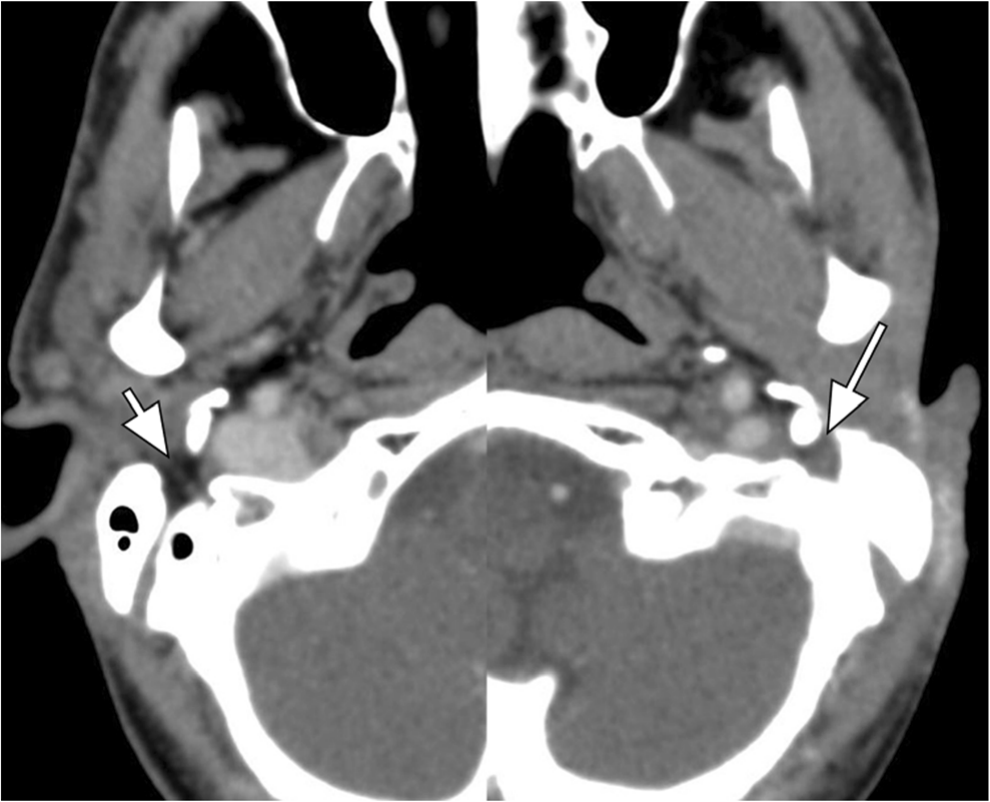
Axial CT composite image shows obliteration of the fat pad at the left stylomastoid foramen (*long arrow*), compared to the normal fat pad on the right (*short arrow*). This finding is suggestive of PNTS along CN VII in particular in a patient with history of skin or parotid malignancy

The facial nerve is comprised of a larger motor root and a smaller sensory root. The motor root provides innervation to the superficial muscles of facial expression as well as the buccinator muscle [14], platysma and the posterior belly of the digastric muscle. The sensory root (called the nervus intermedius) supplies taste sensation to the anterior two-thirds of the tongue as well as general sensation to the ear and adjacent skin. The sensory root also provides parasympathetic innervation controlling secretion of the lacrimal glands, nasal cavity and paranasal sinuses (greater superficial petrosal nerve), as well as the submandibular and sublingual glands (chorda tympani). The facial nerve emerges from the lateral aspect of the pontomedullary junction to course laterally within the anterior superior aspect of the internal auditory canal and subsequently through the otic capsule, along the medial wall of the middle ear cavity, and through the mastoid bone to exit the skull base through the stylomastoid foramen. The stylomastoid foramen contains a prominent fat pad that is best visualised in the axial plane (Fig. 8). The extracranial main trunk of the facial nerve runs anterolaterally into the parotid gland. Within the parotid gland, the facial nerve divides into the following superficial motor branches: temporal, zygomatic, buccal, marginal mandibular and cervical nerves. PNTS along CN VII can cause paresis of the involved facial muscles. Obliteration of the fat pad within the stylomastoid foramen can be detected on non-optimised head, neck and sinus imaging studies and provides an important clue to facial nerve pathology (Fig. 8). Occasionally, denervation atrophy of the superficial muscles of facial expression, buccinator muscle or the posterior belly of the digastric muscle is detected on non-optimised studies.

### Advanced PNTS

PNTS may also occur along nerve branches connecting the trigeminal nerve and the facial nerve. Several earlier publications have described PNTS along these pre-existing neural interconnections. The major known neural communications between these two nerves include the Vidian nerve [15], greater superficial petrosal nerve [16] and auriculotemporal nerve [12]. The focus of our paper is on (easily detectable) signs of PNTS on (non-optimised) CT and MR studies. Detection of PNTS along these interconnections usually require more focused imaging and is therefore not further discussed in this paper.

## Discussion

Obliteration of fat pads and denervation atrophy as outlined in Table 1 are often easily detectable clues for PNTS along the trigeminal or facial nerve even on non-optimised, non-contrast CT and MRI studies. The majority of the fat pads as well as chronic denervation atrophy can be detected on axial CT and MRI studies. Only a few of the fat pads as delineated above and in Table 1 are better seen in the sagittal or coronal plane. The wide utilisation of volumetric CT acquisition allows easy and quick multiplanar reformations on the picture archive computer system (PACS) and is not considered a hindrance or limitation for radiological interpretation. Since almost half of patients with trigeminal or facial nerve pathology are asymptomatic, it is critical that radiologists include the provided list of fat pads (Table 1) into their search pattern even in the interpretation of non-optimised and non-contrasted CT and MRI studies. This is particularly crucial in patients with head and neck malignancies. Early PNTS might still be resectable or easier treatable with radiation therapy, leading to a better patient outcome.

Fat suppressed MR images pose a limitation in detectability of fat pad obliteration by PNTS along the trigeminal or facial nerve as the tumour usually assumes isointensity with the suppressed fat, making it “invisible”. Therefore, non-fat-suppressed MR images are most helpful for the detection of abnormal fat pads. Another limitation to the detection of PNTS is the lack of fatty bone marrow in children and young adults within the mandible around the inferior alveolar canal. In these patients, even contrast-enhanced studies may not be helpful as the red bone marrow usually experiences enhancement similar to neuronal pathology. Fortunately, this age group is unlikely to be affected by malignant trigeminal or facial nerve lesions such as PNTS.

## Summary/conclusions

Obliteration of certain fat pads and chronic denervation of specific muscles represent easily detectable signs of PNTS along the trigeminal or facial nerve even on non-focused imaging studies. The provided “quick search checklist” will facilitate detection of such pathology by the interpreting, even non-subspecialised radiologist leading to earlier diagnosis and treatment especially of malignant neuronal lesions such as PNTS that in turn might lead to improved patient’s outcome.

## Abbreviations

PNTS: Perineural tumour spread
PPF: Pterygopalatine fossa
